# Emerging RAS-directed therapies for cancer

**DOI:** 10.20517/cdr.2021.07

**Published:** 2021-04-08

**Authors:** Michael Conroy, Darren Cowzer, Walter Kolch, Austin G. Duffy

**Affiliations:** ^1^Department of Medical Oncology, Mater Misericordiae University Hospital, Dublin 7, Ireland.; ^2^Systems Biology Ireland, School of Medicine, University College Dublin, Belfield, Dublin 4, Ireland.; ^3^Conway Institute of Biomolecular & Biomedical Research, University College Dublin, Belfield, Dublin 4, Ireland.; ^#^Authors contributed equally.

**Keywords:** Ras, pancreatic, targeted, signalling, isoform, erk, inhibition

## Abstract

RAS oncogenes are the most commonly mutated oncogenes in human cancer, and RAS-mutant cancers represent a major burden of human disease. Though these oncogenes were discovered decades ago, recent years have seen major advances in understanding of their structure and function, including the therapeutic and prognostic significance of diverse isoforms. Targeting of these mutations has proven difficult, despite some successes with inhibition of RAS effector signalling. More recently, direct RAS inhibition has been achieved in a trial setting. While this has yet to be translated to everyday clinical practice, this development carries much promise. This review summarizes the diverse approaches that have been taken to RAS inhibition and then focuses on the most recent developments in direct inhibition of KRAS(G12C).

## INTRODUCTION

Since the discovery of RAS oncogenes as the transforming genes of oncogenic retroviruses^[1,2]^ in the 1960s, great advances have been made in our understanding of their structure and role in human cancer. The three RAS genes, Kirsten rat sarcoma viral oncogene homolog (*K*-*RAS*), neuroblastoma RAS viral (v-ras) oncogene homolog (*N*-*RAS*) and Harvey rat sarcoma viral oncogene homolog (*H*-*RAS)*, are the most commonly mutated oncogenes in human cancer with approximately one-third of all cancers driven by these oncoproteins, including 40%-50% of colorectal cancer and over 90% of pancreatic cancers^[3,4]^. The discovery of oncogenes and the elucidation of intracellular signalling pathways heralded an era of targeted therapy that has greatly improved the outlook for many cancers^[5,6]^. New agents targeting receptor kinases and their downstream mediators demonstrated the ability to stabilise and shrink tumours, with side effects that were frequently milder than those associated with standard cytotoxic chemotherapy. Throughout this enormous paradigm shift in cancer therapeutics, however, RAS stood apart, dominant and seemingly undruggable^[4]^. The development of direct RAS inhibitors proved very challenging. RAS has a high affinity towards GDP and GTP and a lack of deep hydrophobic pockets which would allow binding of small molecules^[7]^. Subtle differences in structure and variable activation of RAS proteins added greatly to the complexity and attention largely focused on downstream inhibition of the transduced signalling pathways^[8]^. In recent years, however, there have been some very promising developments in direct RAS targeting which would suggest that this has real potential as a therapeutic avenue. Here we aim to review current efforts at RAS inhibition in the context of both RAS family biology and the historical efforts which attempted, largely without success, to perturb its role as a major oncogenic driver.

## RAS STRUCTURE AND FUNCTION

**Figure 1:**
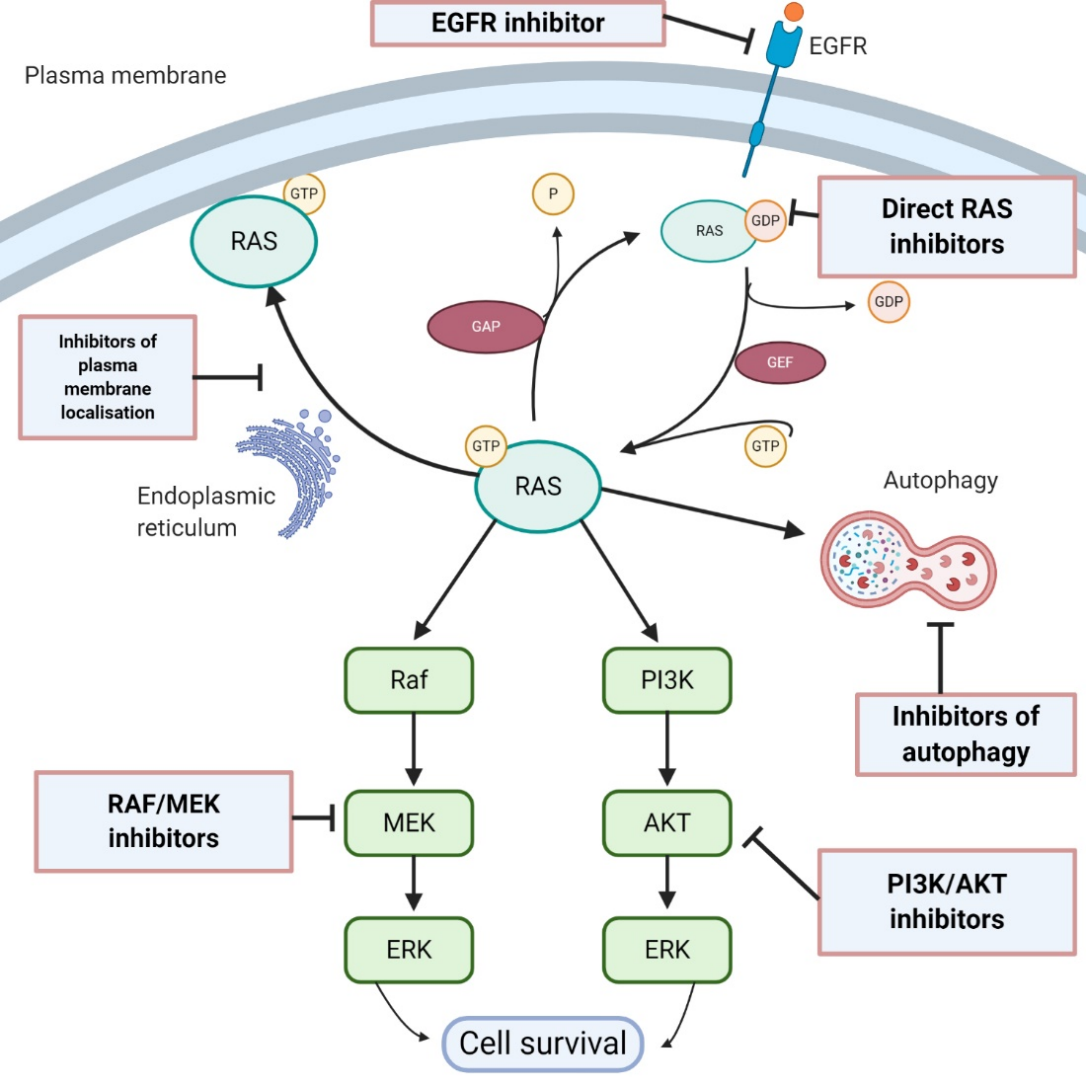
Scheme of the critical role of RAS in biological processes that regulate cell proliferation, survival and autophagy, and potential therapeutic targets (created with BioRender.com)

The RAS superfamily of genes has about 36 members, which encode for 39 proteins^[9]^. Three RAS genes, H-RAS, K-RAS and N-RAS, encode four protein isoforms: H-RAS, K-RAS4A, K-RAS4B and N-RAS. K-RAS4B is the predominant isoform and is referred to simply as K-RAS in this article. RAS proteins are small GTPases, of about 21kD molecular weight, and are monomeric proteins that have a central role in cell differentiation, adhesion, migration, proliferation and survival^[10]^. RAS proteins convey signals from growth factors and extracellular components, and are upstream of signalling pathways including the ERK pathway and the PI3K/mTOR survival pathway. As illustrated in Figure 1, they function as a membrane-bound molecular switch, alternating between an inactive GDP-bound state and an active GTP-bound state. This alternation is mediated by guanine nucleotide exchange factors (GEFs) and GTPase activating/accelerating proteins (GAPs). GEFs are activated by an upstream mitogenic signal, and they in turn cause an inactive RAS to shed its GDP and bind a GTP, which has a 10-fold higher cellular concentration than GDP, thereby becoming activated. This period of activity terminates when the intrinsic GTPase activity of RAS-GTP is enhanced by GAPs, leading to hydrolysis of the bound GTP. In normal cells, a tight equilibrium is maintained between the active and inactive states.

### Pathogenesis

The normal function of RAS, as described above, can be deranged by mutations which unbalance this equilibrium. Single point missense mutations in codons G12 (most commonly), G13 or Q61 are responsible for converting proto-oncogenes to oncogenes. These mutations favour GTP binding and lead to constitutive activation of RAS, with reduction or loss of GTPase activity. These codons are implicated as their amino acid residues are found in the cavity where GTPase catalytic activity operates^[10]^. The consequence of this aberrantly activated RAS is prolonged oncogenic signalling rather than short, controlled bursts of activation^[11]^. There is subsequent activation of downstream signalling molecules such as PI3K, RAF and Rin1. By contrast, mutations in other RAS codons, or any nonsense mutations, are likely to inhibit rather than enhance the activity of RAS, and do not provide a survival advantage. As well as uncontrolled proliferation, the mutagenic RAS oncogene has been implicated in tumour immune resistance by causing intrinsic - as opposed to adaptive - upregulation of programmed death ligand 1^[12]^.

### RAS in cancer

Approximately 25%-30% of cancers contain mutations in one of the RAS isoforms, and they are considered an early genetic event in tumour progression^[4]^. For example, in pancreatic adenocarcinoma, where RAS mutations are virtually ever-present, precursor lesions - pancreatic intraepithelial neoplasia (PanIN) - contain RAS mutations which increase in frequency as they progress stepwise to malignancy. Though generally cooperative with other oncogenes during malignant transformation, these mutations are capable of neoplastic growth in the absence of further genetic abnormalities^[13,14]^. K-RAS is the isoform most frequently mutated and in addition to being almost inevitably mutated in pancreatic cancers, K-RAS mutations are present in approximately half of colorectal cancers and a third of lung cancers^[3]^. H-RAS mutations are found in salivary gland cancers (15% of salivary gland cancers), cervical cancers (9%) and urinary tract cancers (9%). N-RAS mutations are found in melanoma (17%), haematologic malignancies (10%) and thyroid cancers (7%)^[15]^. Not only are RAS mutations implicated in carcinogenesis, but also they are strongly associated with treatment resistance^[16,17]^, and have been shown to be adverse prognostic markers in cancer^[18]^. RAS-mutated colorectal cancer will not benefit from EGFR-directed treatment with cetuximab or panitumumab. Similarly, in lung cancer RAS status has been shown to be an independent predictor for EGFR tyrosine kinase inhibition^[19]^.

## APPROACHES TO RAS INHIBITION

Given the central role of RAS both in carcinogenesis and tumour progression, the RAS oncoprotein is an important therapeutic target. Table 1 provides a summary of current trials of Ras inhibitors. Despite decades of efforts, however, it has proven extremely difficult to synthesise clinically effective direct inhibitors of RAS oncoproteins. This has been attributed to the high affinity of RAS towards GDP and GTP (in contrast to the low affinity of ATP for protein kinases, for example), and lack of deep hydrophobic pockets that would allow the binding of small molecules^[7]^. In addition, given variation in the frequency of isoform mutations within different cancer types, and in the specific mutations involved, there may not be a single effective RAS inhibitor for all RAS-mutated cancers^[4]^. Historical efforts to target RAS which have made it to the clinic could be broadly summarized as those focusing on RAS plasma membrane localisation and those attempting to indirectly block RAS by inhibiting the downstream effector signalling.

**Table 1:** Ongoing RAS inhibitor trials

**Study/Phase**	**Drug**		**Disease**	**Biomarker**	**Clinicaltrials.gov registration no.**
Direct RAS inhibitors					
A phase I/II, study evaluating the safety, tolerability, PK, and efficacy of AMG 510 in subjects with solid tumours with a specific KRAS mutation (CodeBreak 100)	AMG510		Advanced/Metastatic solid tumours	KRAS^G12C^ Mutant	NCT03600883
A Phase 1b, Protocol Evaluating the Safety, Tolerability, Pharmacokinetics, and Efficacy of AMG 510 (pINN) Sotorasib Monotherapy and in Combination with Other Anti-cancer Therapies in Subjects with Advanced Solid Tumours With KRAS p.G12C Mutation (CodeBreak 101)	AMG510 PD1 inhibitor MEK inhibitor SHP2 allosteric inhibitor Pan-ErbB TKI PD-L1 inhibitor EGFR inhibitor Chemotherapy		Advanced/Metastatic solid tumours	KRAS^G12C^ Mutant	NCT04185883
Phase I/II Study of MRTX849 in patients having a KRAS G12C Mutation KRYSTAL-1	MRTX849		Advanced/Metastatic solid tumours	KRAS^G12C^ Mutant	NCT03785249
First-in-Human Study of JNJ-74699157 in participants with Tumours Harboring the KRAS G12C Mutation	JNJ-74699157 (ARS-3248)		Advanced/Metastatic solid tumours	KRAS^G12C^ Mutant	NCT04006301
A Phase I/II study of LY3499446 administered to patients with advanced solid tumours with KRAS G12C mutation	LY3499446 Abemaciclib Cetuximab Erlotinib docetaxel		Advanced/Metastatic solid tumours	KRAS^G12C^ Mutant	NCT04165031
A Study to evaluate the safety, Pharmacokinetics, and activity of GDC-6036 in participants with advanced or metastatic solid tumours with a KRAS G12C mutation	GDC-6036		Advanced/Metastatic solid tumours	KRAS^G12C^ Mutant	NCT04449874
Indirect Targeting of RAS					
SOS inhibitor					
A study to test different doses of BI-1701963 alone and in combined with trametinib in patients with different types of advanced cancer (solid tumours with KRAS mutation)		BI-1701963 BI-3406 trametinib	Advanced/Metastatic solid tumours	KRAS mutations	NCT04111458
SHP2 inhibitors					
Dose escalation of RMC-4630 monotherapy in relapsed/refractory solid tumours		RMC-4630	Advanced/Metastatic solid tumours	Mutations that hyperactivate ERK pathway	NCT03634982
Dose finding study of TNO155 in adult patients with advanced solid tumours		TNO155	Advanced/Metastatic solid tumours	EGFR or KRAS^G12C^ mutations	NCT03114319
Phase Ib study of TNO155 in combination with Spartalizumab or Ribociclib in selected malignancies		TNO155 Spartalizumab ribociclib	Advanced/Metastatic solid tumours	KRAS mutations	NCT04000529
First-in-Human Study of the SHP2 Inhibitor BBP-398 in patients with Advanced Solid Tumours		BBP-398	Advanced/Metastatic solid tumours	MAPK-pathway alterations (excluding BRAF V600X)	NCT04528836
Farnesyltransferase inhibitors					
Phase II study of Tipifarnib in squamous head and neck cancer with HRAS mutations		Tipifarnib	Advanced head and neck squamous cell cancer	HRAS mutations	NCT02383927
RAF inhibitors					
Phase I study of LXH254 in patients with advanced solid tumours harbouring MAPK pathway alterations		LXH-254 Anti-PD-1 antibody	Advanced/Metastatic solid tumours	ERK pathway mutations	NCT02607813
Cobimetinib and HM95573 in Patients with Locally Advanced or Metastatic Solid Tumours		Belvarafenib cobimetinib	Advanced/Metastatic solid tumours	RAS or RAF mutation	NCT03284502
A Phase Ib Study of LXH254-centric Combinations in NSCLC or Melanoma		LXH-254 Trametinib LTT462 Ribociclib	Advanced/Metastatic solid tumours	KRAS or BRAF mutant NSCLC or NRAS mutant melanoma	NCT02974725
Study of the Safety and Pharmacokinetics of BGB-283 (Lifirafenib) and PD-0325901 (Mirdametinib) in Participants with Advanced or Refractory Solid Tumors		BGB-283 PD-0325901	Advanced/Metastatic solid tumours	KRAS mutant NSCLC or endometrial cancer	NCT03905148
MEK inhibitors					
Trametinib and HDM201 in colorectal cancer patients with RAS/RAF mutant and TP53 wild-type advanced/metastatic colorectal cancer I		HDM201 (MDM2 inhibitor) Trametinib	Colorectal cancer	RAS mutant and TP53 wild type	NCT03714958
Atezolizumab and Cobimetinib in Treating Patients with Metastatic, Recurrent, or Refractory Non-small Cell Lung Cancer		Cobimetinib atezolizumab	Advanced/Metastatic solid tumours	KRAS mutation	NCT03600701
Study of MK-8353 + Selumetinib in Advanced/Metastatic Solid Tumors (MK-8353-014)		Selumetinib MK-8353	Advanced/Metastatic solid tumours	none	NCT03745989
Trial of Trametinib and Ponatinib in Patients with KRAS Mutant Advanced Non-Small Cell Lung Cancer		Trametinib ponatinib	Advanced/Metastatic solid tumours	KRAS mutation	NCT03704688
Trametinib and Hydroxychloroquine in Treating Patients With Pancreatic Cancer		Trametinib hydroxychloroquine	Advanced pancreas cancer	none	NCT03825289
Trametinib and Docetaxel in Treating Patients with Recurrent or Stage IV KRAS Mutation Positive Non-Small Cell Lung Cancer		Trametinib Docetaxel	Metastatic NSCLC	KRAS mutation	NCT02642042
ERK inhibitors					
First-in-Human Study of KO-947 in Non-Hematological Malignancies		KO-947	Advanced/Metastatic solid tumours	BRAF, KRAS, NRAS or HRAS mutation	NCT03051035
A Study of LY3214996 Administered Alone or in Combination with Other Agents in Participants with Advanced/Metastatic Cancer		LY-3214996	Advanced melanoma or NSCLC	BRAF or NRAS mutations	NCT02857270
Adoptive Cell therapies					
Administering Peripheral Blood Lymphocytes Transduced with a Murine T-Cell Receptor Recognizing the G12D Variant of Mutated RAS in HLA-A*11:01 Patients		Anti-RAS G12D mTCR	Advanced/Metastatic solid tumours	HLA-A11:01 RAS^G12D ^mutation	NCT03745326
Administering Peripheral Blood Lymphocytes Transduced with a Murine T-Cell Receptor Recognizing the G12V Variant of Mutated RAS in HLA-A*11:01 Patients		Anti-RAS G12D mTCR	Advanced/Metastatic solid tumours	HLA-A11:01 RAS^G12D ^mutation	NCT03190941
Vaccine therapy					
A Study of mRNA-5671/V941 as Monotherapy and in Combination with Pembrolizumab (V941-001)		mRNA-5671	Advanced NSCLC, non-MSI-high CRC, PDAC	HLA-A11:01 and/or HLA-C08:02; KRAS^G12C^, KRAS^G12D^, KRAS^G12V ^or KRAS^G13D ^mutation	NCT03948763
Immunotherapy					
A study of Avelumab, Binimetinib and Talazoparib in patients with locally advanced or metastatic RAS-mutant Solid Tumors I/II		Avelumab Binimetinib Talazoparib	Solid tumours	KRAS or NRAS mutant	NCT03637491
A study of Binimetinib + Nivolumab plus or minus Ipilimumab in patients with previously treated Microsatellite stable metastatic colorectal cancer with RAS mutation I/II		Binimetinib Nivolumab ipilimumab	Colorectal cancer	RAS mutations	NCT03271047

### RAS membrane localisation

In order to carry out their role, RAS proteins must become membrane-bound. This involves a complex series of post-translational modifications. Three enzymatic steps are necessary for RAS to associate with membranes - (1) prenylation of the CAAX box by farnesyltransferase (FTase); (2) cleavage of the terminal AAX residues by RAS converting enzymes RCE1; and (3) methylation of the cysteine residues of the CAAX box by isoprenylcysteine carboxyl methyltransferase ICMT. Farnesylation, the addition of farnesyl groups to RAS, is a critical step in creating a hydrophobic domain in RAS that allows the protein to associate with the plasma membrane, and therefore to be biologically active. Farnesyltransferases (FTases) are the enzymes responsible for this step, and they were an early target in efforts to inhibit RAS function^[20]^. Two farnesyltransferase inhibitors (FTIs), lonafarnib and tipifarnib, were investigated in Phase III trials either as monotherapy or in combination with chemotherapy in a number of different RAS-mutated tumours. Despite Phase I and II clinical trials showing some antitumour activity and low toxicity, no improvement in overall survival was reported in Phase III trials^[21-23]^. One reason for lack of “pan-RAS” efficacy for the FTI class is that K-RAS and N-RAS membrane localisation can be achieved in the absence of farnesyltransferases, via geranylgeranyl transferases. Attempts were made to target these enzymes with geranylgeranyltransferase inhibitors, but these were ineffective and associated with toxicity^[24]^. In contrast, H-RAS is not a substrate for geranylgeranyl transferase and therefore its membrane localization could be suppressed solely by FTIs^[25]^. Tipifarnib has demonstrated preclinical activity against a wide panel of H-RAS-mutated head and neck squamous cell carcinoma xenograft models and is undergoing clinical development in advanced head and neck cancers harbouring activating H-RAS mutations (NCT02383927)^[26]^*. *These efforts highlight the differences between RAS isoforms and the need for a tailored, isoform-specific approach to clinical trial design.

Salirasib is an S-trans, trans-farnesylthiosalicylic acid and a novel oral RAS inhibitor which competes with farnesylated RAS for binding sites on membranes. A recent trial demonstrated encouraging activity in patients with advanced solid tumours including a subset with K-RAS mutations^[27]^. A further target is isoprenylcysteine carboxyl methyltransferase (ICMT), an enzyme at the endoplasmic reticulum which increases RAS membrane affinity. It lacks homology with other protein methyltransferases, which adds to its specificity as a target. While agents that target ICMT have been isolated, and have demonstrated promising results *in vitro* against cancer cell lines, they have not been tested in the clinical setting^[28]^. Palmitoylation, the modification by the fatty acid palmitate, is necessary for the membrane interactions of H-RAS and N-RAS. Depalmitoylation inhibitors and palmitoyl acyltransferases have been described as having activity against RAS, but uncertainty regarding their specificity and concern about off-target effects, have impeded their further clinical development^[4]^. Many other proteins are modified by prenylation and farnesylation to ensure their correct subcellular localization, which makes off-target effects unavoidable even for highly specific inhibitors. Moreover, RAS can also signal from endomembranes (Golgi apparatus, endoplasmic reticulum) in addition to the plasma membrane^[29,30]^. It is unclear how localization inhibitors affect RAS signalling from different subcellular compartments.

### Inhibitors of RAS effector signalling

Given the historic difficulties in directly targeting RAS, many efforts focused instead on inhibiting the downstream signal transduction pathways, either at a single point or as a combined approach targeting different nodal points. RAS effector families are involved in cancer initiation and maintenance, and it was hoped that inhibition of downstream proteins within these pathways could be an effective means of countering RAS-mediated oncogenesis^[28]^. These approaches have largely failed, perhaps mainly due to the inherent complexity and redundancy within these networks, but also due to a lack of specificity in the selection of inhibitors as well as isoform homogeneity within each subpopulation. In addition, the concept of linear pathways is misleading and has given way to that of signalling networks, whereby activated kinases interact via RAS (or other GTPases) with a large variety of signalling molecules resulting in highly interconnected networks.

*EGFR*: There is substantial crosstalk between EGFR tyrosine kinase and RAS^[31]^. With EGFR upstream of RAS, inactivation of these receptor tyrosine kinases can in theory reduce RAS activation, and this linear model explains the lack of clinical activity for EGFR inhibitors in colorectal cancer in the setting of K-RAS or N-RAS-mutant tumours^[32,33]^. This has also been demonstrated experimentally, through activation of the RAS signalling pathway by introduction of an activated K-RAS allele, confirming that as mutated RAS is constitutively active, disruption of signalling from EGFR impairs the therapeutic effect of anti-EGFR monoclonal antibodies^[34]^. Similarly in advanced non-small cell lung cancer (NSCLC), EGFR inhibition is insufficient to produce a response in the setting of RAS-mutant disease^[35]^. It is possible, however, that the specific RAS mutation plays a significant role in determining whether upstream inhibition of EGFR may be effective. We have seen in K-RAS G12D mutation-specific advanced solid tumours that a response can be achieved with pan-ERBB/EGFR inhibitors Afatinib and neratinib^[36,37]^. EGFR and pan-ERBB inhibitors have shown promise in preclinical studies in combination with both direct covalent RAS inhibitors and MEK inhibitors^[37]^. This synergistic effect between EGFR inhibitors and covalent RAS inhibitors appears to be the result of EGFR inhibition leading to a reduced amount of GTP-bound RAS, therefore leaving RAS in the unbound GDP state open to targeting by direct inhibition.

*ERK pathway*: Directly downstream of RAS signaling are the ERK and PI3K signaling pathways^[38]^. Active GTP-bound RAS results in RAF dimerization and phosphorylation, RAF kinase activity and, ultimately, phosphorylation of its substrates MEK1 and MEK2. The terminal kinases of this pathway, ERK1 and ERK2, act as growth promoting transcription factors. The RAS-RAF-MEK-ERK cascade is targeted with RAF kinase inhibitors, ERK inhibitors or MEK inhibitors. Eleven RAF kinase inhibitors have reached clinical evaluation and four are approved for use by the US Food and Drug Administration (FDA). Vemurafenib and dabrafenib are two ATP-competitive RAF inhibitors that are approved for use in BRAF-mutant metastatic melanoma. Further clinical evidence in both lung cancer and colorectal cancer has shown benefit for these agents when BRAF is mutated^[39,40]^. The current agents used in clinical practice, dabrafenib and vemurafenib, act on RAF monomers. However, in RAS-mutant cancer, RAF inhibition has been unsuccessful and the clinical experience has been very negative. The reason for this is that clinically used RAF inhibitors enhance RAF kinase homo-and heterodimerization, leading to the paradoxical activation of ERK signalling^[41]^. Homo- and heterodimerization of the RAF kinases BRAF and CRAF significantly increases their catalytic activities. The binding of RAF molecules to active RAS drives RAF dimerization by inducing conformational changes, dephosphorylation of inhibitory residues, and brings RAF molecules into proximity of each other^[42]^. Due to allosteric interactions between protomers in the RAF dimer, inhibitor binding to the first protomer in a dimer strongly decreases the affinity of the second protomer to the inhibitor. In this constellation the drug-bound RAF protomer allosterically activates the drug-free protomer causing paradoxical pathway activation and drug resistance^[42]^. As oncogenic RAS proteins are effective drivers of RAF kinase dimerization, RAS mutations lead to intrinsic or acquired resistance to RAF inhibitors. Overcoming dimerization-induced resistance to RAF inhibitors could lead to effective anti-RAS therapy^[43]^.

Another interesting drawback to targeting wildtype RAF in RAS-mutant disease is that inhibition of wildtype RAF can paradoxically upregulate the ERK pathway in the setting of RAS mutations leading to downstream phosphorylation of MEK and ERK^[44]^. There are a number of newer agents that target RAF dimers rather than the monomer isoform all of which appear to demonstrate less paradoxical upregulation of the ERK pathway^[45,46]^. Belvarafenib and LXH-254 are pan-RAF inhibitors, effective against the monomer and dimer isoform, which are under clinical evaluation both as monotherapy and in combinations for RAS-mutant advanced solid tumours.

ERK upregulation through MEK appears to be the predominant method of resistance to BRAF directed monotherapy and we have seen both in preclinical and clinical studies that concurrent inhibition of BRAF and MEK can decrease acquired resistance and delay progression^[47]^. In RAS-mutant tumours, MEK inhibition as monotherapy has failed to demonstrate meaningful benefits largely due to the induction of feedback loops similar to those when RAF inhibitors are use in this setting^[48]^. Trametinib, Cobimetinib and Binimetinib are allosteric, non-ATP competitive inhibitors of MEK1 and MEK2, and are used in combination with RAF inhibitors in the treatment of melanoma. A number of trials have failed to show clinical benefit for MEK inhibition in advanced KRAS-mutant pancreatic, colorectal and non-small cell lung cancer^[49-51]^. One possible explanation for this is that inhibition of the ERK pathway induces autophagy, a process of cellular recycling that protects cells from the cytocidal effects of pathway inhibition^[52]^. Pancreatic cancer cells in particular utilize autophagy for growth and as a means of resistance to ERK inhibition^[53]^. Combining downstream MEK inhibition with hydroxychloroquine - an inhibitor of autophagy - displayed synergistic anti-proliferative effects against pancreatic ductal adenocarcinoma (PDA) cell lines and promoted striking regression of PDA xenografts from 2 patients with PDA which was superior to (standard of care) gemcitabine plus nab-paclitaxel chemotherapy. Highly encouraging clinical responses were also seen with this commercially available drug combination and clinical trials are ongoing ^[52,54]^.

*ERK pathway*: With ERK being the final kinase in the RAF-MEK-ERK pathway it would appear to be an attractive target to inhibit in RAS or RAF mutant tumours. Based on previous preclinical studies of the compound SCH-772984, a dual ERK1/2 inhibitor which demonstrated a reduction of phosphorylated ERK in a number of RAS-mutant cancer cell lines, an oral version MK-8353 was developed for clinical testing^[55]^. In a Phase I study, however, with 26 patients with advanced K-RAS or N-RAS mutated tumours, no objective responses were seen. It is now being tested in combination with a MEK inhibitor (selumetinib) and the anti-PD-1 inhibitor pembrolizumab in patients with RAS-mutant cancers. In K-RAS-mutant tumour models, ERK inhibitors such as GDC-0944 have shown efficacy in combination with the MEK inhibitor cobimetinib^[56]^. Phase I studies of the combination were stopped prematurely due to toxicity^[57]^. The ERK inhibitor was studied as monotherapy in a Phase I trial and appeared tolerable^[58]^. In this study, 14 patients had K-RAS-mutant advanced malignancies and of those 4 had stable disease and 10 had progression. ERK pathway suppression detected with NanoString gene expression was observed more commonly in those with BRAF-mutant tumours compared to those with KRAS-mutant tumours.

Ulixertinib is another ATP competitive ERK1/2 inhibitor that has shown clinical efficacy in N-RAS-mutant melanoma^[59]^. This in combination with nab-paclitaxel has been examined in a Phase 1 study in patients with advanced pancreas cancer and results are awaited (NCT02608229). Other studies are underway examining its role as monotherapy and in combination with other agents in patients with genetic alteration in the ERK pathway (NCT03698994, NCT04145297). LY-3214996 is a selective inhibitor of ERK1 and ERK2^[60]^. Unfortunately, however, in a Phase I study of this agent, only one patient with advanced RAS-mutant cancer had stable disease and the remaining patients had progressive disease^[60]^. KO-947, similarly a selective ERK1/2 inhibitor, demonstrated potent and sustained reduction in phosphorylated ERK *in vitro* in RAS-mutant cell lines^[61]^. This is now in clinical studies for patients with advanced RAS- or RAF-mutant tumours (NCT03051035).

*PI3K pathway*: The other major target of RAS effector signalling is the PI3K-AKT-mTOR pathway. PI3K is implicated in RAS-dependent cancer initiation and maintenance. While there are many inhibitors of this signalling pathway under investigation^[62]^, they have demonstrated little activity as monotherapy in RAS-mutant cancers. KRAS and BRAF mutations are predictive of resistance to mTOR inhibition^[63]^. Moreover, inhibition of mTOR may lead to the upregulation and activation of MEK-ERK pathways. Therefore, the dual inhibition of the PI3K and the ERK pathway would appear to be a reasonable therapeutic target. With this in mind combination strategies have been explored in RAS mutant disease using both mTOR inhibitors along with RAF/MEK inhibitors^[64]^. Direct PI3K inhibitors have also been trialled with MEK inhibitors. However, in clinical trials, this combination was both toxic and only minimally efficacious^[65]^.

AKT, which plays a key role in the activation of mTOR following its interaction with one of the 3 main PI3Ks, is amplified in the setting of RAS-mutant cancers^[66]^. Unfortunately no AKT inhibitors are approved for use in clinical practice. The evaluation of AKT inhibition with MEK inhibitors is under investigation, but similar challenging side effect profiles have been seen to that of combined mTOR and MEK inhibition^[67]^.

### Direct inhibitors of RAS

Attempts at direct RAS inhibition have been hampered by numerous challenges. Firstly, the activity of RAS is tightly governed by GEFs and GAPs, which control the high-affinity, picomolar interactions between GDP, GTP and RAS. Putative inhibitors struggle to overcome this affinity. Secondly, there are structural challenges. An effective inhibitor would typically require a deep hydrophobic pocket for binding. These pockets were long understood to be absent, although recent research demonstrates that they may arise dynamically as RAS goes through the GDP/GTP cycle^[68,69]^. Finally, there are selectivity and toxicity challenges. The “switch region”, which changes conformation upon GTP binding and recruits effector proteins, is highly conserved across other G-proteins in the body. Therefore, any agents which target this region in RAS, would be associated with risk of toxicity elsewhere.

A number of agents investigated were compounds that competed directly with GDP for the nucleotide binding site of RAS^[70]^ and compounds that bind to RAS at the RAF binding site and inhibit RAS/RAF complex formation^[71]^. However, none were sufficiently potent to be considered for further investigation. More recently, however, direct targeting of RAS has been achieved for the G12C K-RAS mutation. This has been realised through the development of a small molecule inhibitor that binds covalently to the cysteine residue that results from the specific G12C mutation and has been shown to inhibit oncogenic RAS^[72,73]^. The inherently reactive nature of cysteine which is found at codon 12 of K-RAS(G12C) can be exploited for covalent small molecule inhibitors and the idea of targeting cysteine is one that is commonly exploited in drug discovery^[74]^. Another important feature of targeting this cysteine is that wild-type K-RAS lacks the cysteine in the active site unlike the mutant K-RAS(G12C).

Ostrem *et al*.^[73]^ initially identified this novel allosteric binding pocket behind switch II referred to as the switch-II pocket, which led to the development of the first compounds to irreversibly target G12C. These compounds bind to K-RAS(G12C) in the GDP-bound inactive state, blocking SOS-catalysed nucleotide exchange and ultimately inhibiting K-RAS(G12C) association with RAF^[73]^. This switch II pocket is present in the GDP-bound inactive form of K-RAS only. Therefore, targeting of G12C needs to occur in the GDP-bound state. K-RAS(G12C) in its steady state is in the active GTP-bound state, but the presence of a high level of GTPase activity leaves it open to covalent attack^[75]^. The identification of this pocket led to a search for covalent inhibitors of K-RAS. The first was a molecule ARS-853^[76]^, and its development led to the proof of concept that this specific isoform of K-RAS could be targeted using a covalent inhibitor.

A number of advances in the area ultimately led to the development of AMG510 (sotorasib)^[73]^. This particular G12C inhibitor has succeeded where others had not as its potency and selectivity was optimised through an interaction with a previously unexploited groove His95^[77]^. Preclinical studies have demonstrated responses and regression of K-RAS mutant tumours treated with AMG510. The results of the Phase I study of sotorasib (AMG510) demonstrated promising anticancer activity in patients with advanced solid tumours harbouring K-RAS(G12C) mutations. 129 patients were treated on the dose escalation study, 59 with NSCLC, 42 with colorectal cancer and 28 with other solid tumours. Sotorasib appeared to be well tolerated with 11.6% of grade 3 or 4 toxicity. 32.2% of the NSCLC had an objective response with a total of 88.1% having a response or stable disease. The median progression free survival was 6.3 months. In the colorectal cohort 7.1% had a confirmed response with 73.8% having a response or stable disease with a median PFS of 4 months^[78]^. This study represented the first clinical trial demonstrating objective response to direct KRAS inhibition. On December 8, 2020 the FDA granted Breakthrough Therapy designation for its investigational K-RAS(G12C) inhibitor, sotorasib, for the treatment of patients with locally advanced or metastatic NSCLC with K-RAS(G12C) mutations, as determined by an FDA-approved test, following at least one prior systemic therapy. Preclinical studies have also demonstrated that sotorasib was able to clear colon cancer from mice when given in combination with checkpoint inhibitors^[79]^. There is a Phase 1/2 study under way of sotorasib in solid tumours which will include a combination arm of sotorasib with an anti(PD-1/L1) (NCT03600883).

Adagrasib (MRTX849) is an additional agent under investigation in this field. It is a potent, highly selective inhibitor of KRAS(G12C)^[80]^. A Phase 1/2 study of adagrasib monotherapy in patients with pretreated NSCLC demonstrated an overall response rate of 45% and a high disease control rate^[81]^. This multiple-expansion-cohort trial is ongoing, investigating the use of adagrasib combined with pembrolizumab in NSCLC, afatinib in NSCLC or cetuximab in colorectal cancer. Research on this agent in nonclinical models has also demonstrated mechanisms of resistance, including KRAS nucleotide cycling and pathways that induce feedback reactivation or bypass KRAS dependence^[80]^.

K-RAS(G12C) mutations only account for a small proportion of KRAS mutations that are found in cancer and are primarily found in lung cancer. As these irreversible allosteric inhibitors block RAS signalling by exclusively binding to the cysteine residue that results from the specific mutation, this limits their application to the particular allele they target. To target KRAS(G12D) and KRAS(G12V) different approaches are needed as these mutants lack the cysteines needed in the active state.

Attempting to design and develop drugs specifically targeting each individual RAS mutation would be extremely challenging and time-consuming, so direct targeting of ligand binding sites conserved on all RAS proteins (KRAS4A, 4B, NRAS and HRAS) has been thought to be one potential method of inhibiting RAS across all mutation and tumour types. *In vitro* studies have shown that Compound 3144, a molecule that binds a conserved residue Asp38 in switch-I, can block RAS effector binding^[82]^. This compound suppresses the growth of KRAS(G13D) tumours *in vivo*. A major concern, however, is that pan-inhibition of RAS potentially may lead to considerable toxicity as normal cellular function is reliant on RAS signalling in non-cancerous cells^[83]^. Models by which the deletion of all three RAS isoforms is carried out are not compatible with life, and therefore a pan inhibition of RAS in humans is likely to result in significant off-target toxicity. Nevertheless, it has been suggested that if such a compound were optimised for greater potency and specificity, this would be a viable approach^[82]^.

Nucleotide exchange inhibition: In order to cycle between active GTP-bound and inactive GDP-bound states, RAS possesses intrinsic guanine nucleotide exchange and GTP hydrolysis activities. This cycling and exchange are accelerated by GEF and by GAPs, both of which change the activation state of RAS through covalent modifications. Upon activation of GEFs, nucleotide binding is destabilized and GDP is released. As GTP is much more prevalent than GDP in the cell, this loss of GDP leads to a transient formation of RAS-GTP, the active state^[4]^. GTP binding and activation of RAS leads to conformational changes in it, allowing it to bind effectors in RAS-binding domains. Mutations in RAS that are relevant to cancer usually lead to RAS permanently in the GTP bound active state. Efforts have been made to block this nucleotide exchange in an attempt to stop RAS transitioning to the active GTP bound form.

An alternative to blocking the direct nucleotide site is to inhibit the proteins that regulate the nucleotide exchange process. Normal RAS activation requires nucleotide exchange, processing, membrane localization and effector binding. Targeting any of these steps can be used to indirectly inhibit RAS. GEFs are responsible for releasing GDP from RAS allowing it to be replaced by GTP and ultimately leading to activation of RAS. In mammals, three families of RAS-specific GEFs exist: SOS, RASGRF1/Cdc25Mm and GRP/Cal-DAG-GEF^[84]^. Of these, SOS is the best known and studied of the RAS-specific GEFs and this has led to the development of a number of strategies and attempts to block it and ultimately inhibit RAS. SOS1 binds RAS at its catalytic binding site and thereby promotes exchange of GDP for GTP. RAS-GTP can also bind at an allosteric site on SOS1 to enhance GEF activity^[85]^. Genetic inactivation of SOS1 has been shown to decrease the survival of RAS-mutant tumor cells, but not in RAS wild type cells that are not reliant on RAS signaling^[86]^. Inhibition of SOS1 has been thought of as an attractive mechanism of RAS inhibition over direct RAS inhibitors as it does not appear to depend on targeting specific mutations.

BI-3406 is a potent and selective SOS1:K-RAS interaction inhibitor that potently decreased the formation of GTP-bound RAS and reduced cell proliferation of RAS-driven cancers both *in vitro* and *in vivo*. This orally bioavailable agent appears to reduce RAS-GTP level and inhibits ERK pathway signaling, thereby limiting the growth of tumor cells driven by RAS. Whilst most RAS variants appear to show reduction in cell proliferation when exposed to BI-3406, certain variants appear less sensitive. Mutations in codon 61 due to the resultant molecular conformation appear to have low intrinsic GTPase activity and are subsequently less sensitive to SOS1 inhibition^[87]^. Initial studies of SOS1 inhibitor BI-3406 suggest that it may benefit as many as 80%-90% of RAS-driven cancers^[88]^.

SOS1 is phosphorylated by ERK, a kinase in the ERK pathway downstream of RAS, ultimately leading to the reduction of its GEF activity. It is thought that efforts to treat RAS-driven cancers with MEK inhibitors have failed in part as inhibition of MEK reduces the activity of ERK1/2, resulting in the release of a negative feedback loop, thus increasing the activity of SOS1-dependent formation of GTP-bound RAS. Combination therapy of a MEK inhibitor with BI-3406 blocks this negative feedback by reducing levels of phospho-MEK and phospho-ERK leading to sustained pathway inhibition and potentiating the benefit of SOS1 inhibition. A Phase I clinical trial of this combination to assess safety, tolerability and preliminary efficacy, as well as another SOS1 inhibitor, BI-1701963 in combination with Trametinib is ongoing (NCT040111458).

Although much less defined than SOS, another protein involved in the nucleotide exchange process is SHP2. SHP2 is a non-receptor protein tyrosine phosphatase that is required for the full activation of the ERK pathway^[89]^. Mutations in PTPN11, which encodes SHP2, cause “rasopathies” and are found in about 50% of patients with Noonan syndrome^[90]^. Although not fully defined, SHP2 appears to act as a scaffold protein, binding GRB2 and SOS1 in close proximity to RAS and ultimately thereby increasing RAS nucleotide exchange^[91]^. Research has explored the role of SHP2 inhibitors in the treatment of various cancers. In the preclinical setting, the allosteric SHP2 inhibitor SHP099 has been shown to inhibit myeloid leukaemia cell lines^[92]^, and to elicit a response in colorectal cancer cell lines^[93]^. Of note, in the colorectal cancer cells, these responses seemed limited to cells that were sensitive to lapatinib, and therefore dependent on EGFR signalling. By contrast, RAS- or BRAF-mutant cells were generally resistant to SHP099.

## CONCLUSION AND FUTURE DIRECTIONS

Effective and safe inhibition of RAS was considered a holy grail for cancer researchers decades ago, and it remains so today. While extraordinary advances have been made in our understanding of RAS and carcinogenesis - as well as methods of targeting its downstream effectors - the direct inhibition of RAS does not yet have a role in everyday practice. However, our deeper understanding of molecular pathogenesis is the foundation on which that future progress will be built, and effective treatments are now close.

The unmet need for proven therapies in this setting is clear, given the prevalence of RAS mutations in common cancers, many of which are highly fatal. Our recognition of the structure and clinical significance of diverse RAS isoforms represents a major step forward, of relevance to all future efforts at RAS targeting. Their importance is clear when considering candidate inhibitors of RAS plasma membrane localization, which are now reaching the clinic. Even individual isoforms, like K-RAS, have a range of possible mutations with different responses to targeted therapies^[94]^. Therefore, those designing clinical trials should carefully consider the need for isoform and mutation-specific approaches.

The development of inhibitors of RAS effector signalling, which are in widespread clinical practice, is an example of the concrete achievements of research in this area. This success has been tempered over time by failures, many of which can be attributed to misconceptions of RAS signalling pathways as unidirectional and linear. A more nuanced appreciation of these as delicately balanced signalling networks can help researchers refine their approach, and may warrant increased focus on drug combinations, as seen in trials involving inhibitors of MEK and autophagy^[52]^.

Possibly showing greater promise than any of these initiatives, however, is efforts at direct targeting of RAS. The past 12 months has seen breakthroughs in this field that have been sought for years, vindicating decades of basic scientific endeavour. Successes here belie outdated dogma describing RAS as “undruggable”, demonstrating that drug exposure associated with preclinical efficacy can be achieved safely in patients^[95]^. However, these studies also raise questions - why did response rates to Sotorasib vary between cancers despite identical mutations, and what provoked early progression in some patients after an initial response? It is suggested that tumour heterogeneity or driver mutations alternative to K-RAS(G12C) may be the key to understanding this and that, as with inhibition of RAS effector signalling, drug combinations may be the next step^[78]^. In addition, optimizing monotherapy by developing inhibitors with higher affinity for inactive KRAS(G12C) or the use of pulsatile therapy has been suggested as approaches to maximise therapeutic effect^[96]^.

In summary, a more textured understanding of RAS pathogenesis emerging from decades of basic scientific research has led to a more refined approach to RAS inhibition, which is now beginning to bear fruit. The coming months and years will hopefully take these efforts “over the line” to the routine use of effective therapeutics in the clinic setting, but it will be a long time before we have fully tapped the potential of RAS inhibition.

## DECLARATIONS

### Copyright

© The Author(s) 2021.
